# Twinkle-associated familial parkinsonism with Lewy pathology: Cause or predisposition?

**DOI:** 10.1212/WNL.0000000000010674

**Published:** 2020-10-06

**Authors:** David P. Breen, David G. Munoz, Anthony E. Lang

**Affiliations:** From the Centre for Clinical Brain Sciences (D.P.B.), University of Edinburgh; Anne Rowling Regenerative Neurology Clinic (D.P.B.), University of Edinburgh; Usher Institute of Population Health Sciences and Informatics (D.P.B.), University of Edinburgh, Scotland; Department of Laboratory Medicine (D.G.M.), St Michael's Hospital, Toronto, Canada; Edmond J. Safra Program in Parkinson's Disease and the Morton and Gloria Shulman Movement Disorders Clinic (A.E.L.), Toronto Western Hospital; Department of Medicine (A.E.L.), University of Toronto; and Krembil Research Institute (A.E.L.), Toronto Western Hospital, Ontario, Canada.

Mitochondrial dysfunction is a recognized cause of autosomal recessive Parkinson disease (PD) and may contribute to idiopathic disease.^1^ Twinkle protein is a DNA helicase coded by the *C10orf2* gene, which, along with polymerase gamma and other proteins, is responsible for regulating mitochondrial replication. Heterozygous *C10orf2* mutations are a recognized cause of chronic progressive ophthalmoplegia (CPEO) and other neurologic manifestations, but their relationship with parkinsonism is unclear. Here, we report a case (along with postmortem examination findings) of familial parkinsonism associated with a heterozygous mutation in *C10orf2*, alongside reviewing previously published cases.

## Case report

A 61-year-old man was referred to our clinic with an 18-month history of left leg dragging and left arm motor dysfunction (e.g., difficulty putting hand in pocket). Family members commented that he had a softer voice and reduced facial expression. Nonfatigable bilateral eyelid ptosis was present on his driving license 3 years earlier. His mother had been diagnosed with PD: she presented with shuffling gait and poor balance in her early 60s, responded well to levodopa but developed peak-dose dyskinesias, and died aged 79 years.

Examination confirmed bilateral ptosis (palpebral fissures 8 mm vertically and normal levator excursion) and a mild complex ophthalmoplegia. There was evidence of parkinsonism (predominantly affecting the left hemibody), which improved with levodopa (Video 1).

10.1212/010674_Video_1 Video 1 Examination took place a few years after he presented to our clinic. He was taking levodopa (total daily dose 800 mg), and his parkinsonian signs were less marked. The video shows evidence of bilateral ptosis with mild restriction of eye movements (particularly upgaze). There was no improvement on vestibulo-ocular reflex testing (not shown). There was very mild bradykinesia in the limbs, along with mild choreiform movements. Gait was fairly normal apart from a slight lean to the right and mild impairment on tandem walking.Download Supplementary Video 1 via http://dx.doi.org/10.1212/010674_Video_1

MR brain scan showed patchy small vessel ischemic changes in the pons but no other abnormalities. Single-fiber electromyogram was abnormal with a mean jitter duration 47 microseconds (normal <36). Acetylcholine receptor antibodies were negative. *POLG* testing revealed no pathogenic mutations. Muscle biopsy was booked, but the patient did not attend. A provisional diagnosis of PD with CPEO was made, although the possibility of a unifying etiology related to mitochondrial dysfunction was considered.

Over the next few years, additional medications (selegiline, entacapone, and pramipexole) were sequentially added due to the development of motor fluctuations including mild generalized dyskinesias. Around 7 years after diagnosis, he began to develop nonmotor complications including cognitive decline, falls (ultimately requiring a walking frame), neuropsychiatric symptoms (visual hallucinations and paranoia), and swallowing difficulties. These progressively worsened despite medication alterations (pramipexole and selegiline stopped; rivastigmine and quetiapine started). He was eventually admitted to a nursing home and died approximately 10 years after diagnosis due to a presumed aspiration pneumonia.

Just before his death, further genetic testing revealed a heterozygous variant in the *C10orf2* gene on chromosome 10 (c.908 G>A, p.[Arg303Gln]). Bioinformatic analysis predicted that this variant was pathogenic and had a very low allele frequency on gnomAD (0.0012%). PD gene panel testing identified no other clinically relevant variants.

Postmortem examination revealed severe, patchy neuronal cell loss in the substantia nigra with evidence of limbic (transitional) stage Lewy body disease according to the Montine classification (figure).^2^ In the substantia nigra, alpha-synuclein immunostains labeled Lewy bodies and Lewy neurites, as well as diffusely filled perikarya. Lewy bodies were also present in the locus coeruleus, periaqueductal gray matter, basal temporal neocortex, and cingulate gyrus (figure, C, D, H); but sparse in other regions of the neocortex. There were no abnormalities in the cerebellum. There was no significant deposition of tau, beta-amyloid, or TDP-43 proteins.

**Figure:**
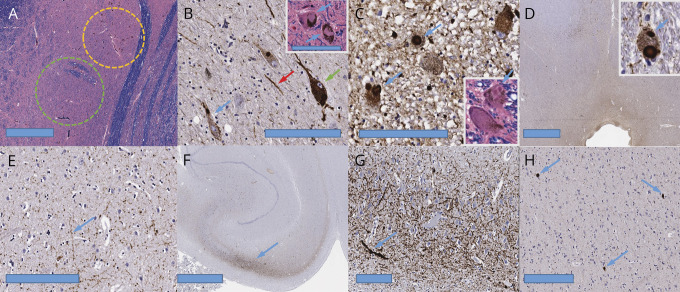
Neuropathology of Twinkle-associated parkinsonism (A) Low-power view of the substantia nigra showing patchy loss of neurons, with areas of complete loss (green circle) contrasting with others of partial preservation (yellow circle), bar 800 µm. Luxol fast blue (LFB) staining on hematoxylin and eosin. (B) Substantia nigra immunostained for alpha-synuclein showing Lewy bodies (blue arrows), Lewy neurites (red arrow), and neurons with cytoplasm diffusely filled with alpha-synuclein (green arrow), bar 200 µm. The inset shows Lewy bodies (blue arrows) in 2 neurons on LFB staining, bar 100 µm (applies to all insets). (C) Locus coeruleus immunostained for alpha-synuclein, showing labeled neurons with Lewy bodies (blue arrows), bar 200 µm. The inset shows Lewy bodies on LFB staining (blue arrows). (D) Periaqueductal gray (aqueduct at center bottom) immunostained for alpha-synuclein showing labeled neurons with Lewy bodies (inset, blue arrow), bar 2 mm. (E) Putamen immunostained for alpha-synuclein showing Lewy neurites (blue arrow), bar 200 µm. (F) Hippocampus immunostained for alpha-synuclein showing labeling in the CA2 sector (blue arrow), bar 2.5 mm. (G) Higher power view of the alpha-synuclein immunostained CA2 area of the hippocampus showing predominantly horizontally oriented Lewy neurites (blue arrow), bar 200 µm. (H) Temporal neocortex immunostained for alpha-synuclein showing scattered cortical Lewy bodies (blue arrows), bar 200 µm.

## Discussion

We propose that heterozygous *C10orf2* mutations may be a rare cause of parkinsonism and should be considered in patients with a positive family history and/or other features of a mitochondrial disorder (e.g., CPEO). We searched the literature and found 8 previously reported cases of parkinsonism associated with heterozygous *C10orf2* mutations (table).^3–7^ They are unlikely to be a major contributor to overall PD heritable risk; indeed, *C10orf2* does not appear as a risk loci on the most recent meta-analysis of genome-wide association studies.^8^

**Table:**
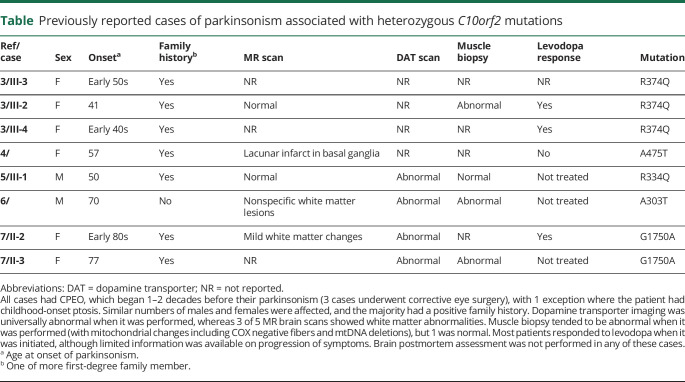
Previously reported cases of parkinsonism associated with heterozygous *C10orf2* mutations

We cannot exclude the possibility that the association may be a coincidence, especially because we were unable to perform segregation analysis. Unfortunately, only fixed brain tissue was available for the postmortem examination, which precluded molecular analysis (such as mtDNA deletion load). In the single reported autopsy case of a patient with heterozygous *C10orf2* mutation, there was also significant loss of substantia nigra neurons (although the patient did not have clinical evidence of parkinsonism), but no Lewy bodies were present (unlike our case).^9^ We hope that neuropathologic analysis of future cases will help to determine the precise pathologic underpinnings of parkinsonism in heterozygous *C10orf2* mutation carriers.

Twinkle protein is important for maintaining mtDNA integrity. In a mouse model expressing mutant Twinkle, there was accelerated accumulation of mtDNA deletions and loss of TH-positive neurons (leading to motor impairment).^10^ Patients with biallelic *C10orf2* mutations typically present with severe and complex neurologic phenotypes (e.g., infantile-onset spinocerebellar ataxia, epilepsy, sensory polyneuropathy, Perrault syndrome, and adult-onset mitochondrial myopathy) alongside systemic features, but not parkinsonism. The classical pathology in these patients includes severe neuronal loss in the substantia nigra in the absence of Lewy bodies or alpha-synuclein deposition, often accompanied by degeneration of the cerebellar-dentato-olivary system. Further studies are required to explain why most patients with biallelic *C10orf2* mutations do not exhibit parkinsonism despite demonstrating severe substantia nigra neuronal loss (which also occurs with biallelic *POLG* mutations).^9^

Heterogeneous neuropathology is a recognized feature of genetic PD associated with mitochondrial dysfunction. A review of autopsy findings in 18 homozygous or compound heterozygous *Parkin* cases found Lewy bodies in only 6 patients, despite evidence of neuronal loss in the substantia nigra in all cases.^11^ In the 2 postmortem cases of biallelic *PINK1* mutations, Lewy bodies were present in one^12^ and absent in the other.^13^ The role of heterozygous *Parkin* and *PINK1* mutations is controversial; however, it is intriguing that the limited number of autopsy studies has shown diffuse Lewy bodies in both groups. This may support the concept of genetic predisposition to PD by the mitochondrial dysfunction caused by these heterozygous states, which is in line with a recent report showing greater Lewy body pathology in older patients with mitochondrial dysfunction due to a range of nuclear and mtDNA genetic defects.^14^
